# Linc00475 promotes the progression of glioma by regulating the miR‐141‐3p/YAP1 axis

**DOI:** 10.1111/jcmm.16100

**Published:** 2020-12-18

**Authors:** Mingjun Yu, Bolong Yi, Wen Zhou, Wei Gong, Gang Li, Shijia Yu

**Affiliations:** ^1^ Department of Neurosurgery Shengjing Hospital of China Medical University Shenyang China; ^2^ Gamma Knife Center, Shengjing Hospital of China Medical University Shenyang China; ^3^ Key Laboratory of Neuro‐oncology in Liaoning Province Shenyang China; ^4^ Department of Pain Management Dalian Municipal Central Hospital Dalian China; ^5^ Exprimental Research Center Shengjing Hospital of China Medical University Shenyang China; ^6^ Department of Neurology Shengjing Hospital of China Medical University Shenyang China

**Keywords:** ceRNA, glioma, linc00475, miR‐141‐3p, prognosis, YAP1

## Abstract

Glioma is the most prevalent and lethal primary brain tumour. Abundant long non‐coding RNAs ( lncRNAs) are aberrant and play crucial roles in the oncogenesis of glioma. The exact functions of linc00475 in glioma remain blurred. Here, we analysed the expression levels of linc00475 by qRT‐PCR and discovered that linc00475 was up‐regulated in glioma and predicted a poor prognosis in patients with glioma. Besides, inhibiting linc00475 restrained the progression of glioma in vitro and in vivo. Further experiments confirmed that linc00475 regulated the progression of glioma by acting as a sponge for miR‐141‐3p. Moreover, we detected the binding sites of linc00475 and miR‐141‐3p, the YAP1‐ 3′UTR and miR‐141‐3p by luciferase reporters. The rescue assays confirmed that inhibiting linc00475 restrained the progression of glioma through the miR‐141‐3p/YAP1 pathway. Collectively, our research demonstrates the key roles of linc00475 in glioma, which could be a promising therapeutic target.

## 1 INTRODUCTION

Glioma is the most prevalent and aggressive malignant primary brain tumour. Although many therapeutic advances have emerged, glioma remains one of the deadliest of all kinds of cancer due to aggressive cell proliferation, migration and invasion.^1^, ^2^ Therefore, it draws great attention to explore the underlying mechanisms and effective therapeutic targets in glioma.

During the last decade, long non‐coding RNAs (lncRNAs) have been identified as significant regulators in tumorigenesis and development by many studies. For instance, lncRNA DSCR8 elevates in hepatocellular carcinoma, and progression is promoted by activating the Wnt/β‐catenin signalling pathway.^3^ LncRNA NFAT5 and LSINCT5 are up‐regulated in glioma and promote the proliferation of glioma.^4^, ^5^ Linc00475 is located on chromosome 9q22.31 and has nine transcripts. Recently, linc00475 has been proposed to be associated with the overall survival of glioblastoma multiforme patients, while the underlying molecular mechanisms of linc00475 in glioma remain to be addressed.^6^

Compelling studies have demonstrated that many lncRNAs could function as competing endogenous RNAs (ceRNA) to bind with specific microRNAs (miRNAs). MiR‐141‐3p plays suppressive roles in hepatocellular carcinoma, osteosarcoma, nasopharyngeal carcinoma and breast cancer.^7^, ^8^, ^9^, ^10^ In addition, miR‐141‐3p is down‐regulated and restrains the tumorigenesis of glioma cells.^11^ Although the anti‐oncogenic role of miR‐141‐3p on glioma has been reported, the mechanistic details are limited.^12^

The Hippo pathway is involved in the regulation of organ development and the progression of tumours. Yes‐associated protein 1 (YAP1), a significant downstream effector of the Hippo pathway, has emerged as a key transcriptional factor promoting the proliferation of cancer.^13^ In addition, high YAP1 expression glioma is more aggressive and is associated with poor outcomes.^14^, ^15^ It has been recently reported that miR‐141‐3p limits the proliferation of stem cells in apical papilla by regulating YAP (YAP1).^16^ Nevertheless, whether YAP1 is the target of miR‐141‐3p in glioma is poorly defined.

In the present study, we revealed that linc00475 is amplified in glioma and was correlated with a poor prognosis. We analysed the roles of linc00475 in the biological function of glioma in vitro and in vivo. Moreover, we validated that linc00475 exerts its oncogenic function by binding with miR‐141‐3p, abolishing the decrease of YAP1 expression, and activating the Hippo pathway. Taken together, the linc00475/miR‐141‐3p/YAP1 axis provides a promising target for glioma therapy.

## 2 MATERIALS AND METHODS

### 2.1 Tissue specimens

Forty glioma tissues and 15 normal brain tissues (NBTs, from injury patients) were collected from glioma patients at Shengjing Hospital between October 2011 and September 2014. The clinical characteristics of the patients, including age, pathological grade, gender and the date of death from glioma are displayed in Table S1. All patients provided informed consent, and this study was approved by the Ethics Committees of Shengjing Hospital (No. 2016PS366K).

### 2.2 Cell culture and reagents

Authenticated U87 and U251 glioma cell lines as well as HEK293T cells were gained from the Chinese Academy of Medical Sciences (Beijing, China) and routinely cultured in DMEM/high glucose medium with 10% heat‐inactivated FBS (Life Technologies). Normal human astrocytes (NHA) were purchased from ScienCell Research Laboratories and cultured in RPMI‐1640 medium with 10% FBS. All cells were maintained at 37°C in 5% CO_2_.

### 2.3 Cell transfection

The assays were performed using the Lipofectamine 3000 reagent (Invitrogen) as described previously.^17^ Sh‐linc00475 (shRNA#1 and shRNA#2), pre‐miR‐141‐3p, anti‐miR‐141‐3p, YAP1 and their negative controls (sh‐NC, pre‐NC and anti‐NC) were purchased from GenePharma. The sh‐linc00475, pre‐miR‐141‐3p, anti‐miR‐141‐3p sequences and their negative controls are listed in Table S2.

### 2.4 Real‐time PCR

The PCR assay was carried out as the protocols depicted previously.^18^ Briefly, total RNA from tissues or cells was extracted using Trizol reagent. The PCR assay was performed on an ABI 7500 system. GAPDH or U6 was employed as the endogenous control for linc00475 or miR‐141‐3p, respectively. The sequences of primers were listed in Table 1.

**Table 1:** Primers for qPCR

Gene	Primers
linc00475	Forward: 5′‐TGTAGTCGGCTGGCTGAGGTC‐3′
	Reverse: 5′‐CCTAAGTGTCGGCTGTGCATGG‐3′
GAPDH	Forward: 5′‐AAATCCCATCACCATCTTCCAG‐3′
	Reverse: 5′‐TGATGACCCTTTTGGCTCCC‐3′
linc00475	Forward: 5′‐TGTAGTCGGCTGGCTGAGGTC‐3′
	Reverse: 5′‐CCTAAGTGTCGGCTGTGCATGG‐3′
miR‐141‐3p	Forward: 5′‐GCGCGTAACACTGTCTGGTAA‐3′
	Reverse: 5′‐AGTGCAGGGTCCGAGGTATT‐3′
U6	Forward: 5′‐CTCGCTTCGGCAGCACA‐3′
	Reverse: 5′‐AACGCTTCACGAATTTGCGT‐3′

### 2.5 Western blot analysis

The Western blot analysis was undertaken as our previous study.^19^ The primary antibodies as below: YAP1 (1:1000, abcam), GAPDH (1:1000, Santa Cruz Biotechnology), MMP2 (1:1000, abcom).

### 2.6 Dual‐luciferase reporter assays

The linc00475 and miR‐141‐3p binding sequences were predicted by the bioinformatics Lncbase (http://carolina.imis.athena‐innovation.gr/).^20^ The YAP1‐3′UTR and miR‐141‐3p binding sequences were predicted by ENCORI bioinformatics (http://starbase.sysu.edu.cn/).^21^ The wild‐type and corresponding mutant sequences of linc00475 and the YAP1‐3′UTR were cloned into the pmirGLO Dual‐Luciferase Vector (linc00475‐wt or linc00475‐mut; YAP1‐3′UTR‐wt or YAP1‐3′UTR‐mut; GenePharma). Then, HEK293T cells were co‐transfected with the pmirGLO vector and miR‐141‐3p agomir, respectively., Luciferase activity was detected after 48 hours, following the manufacturer's instructions (Promega).

### 2.7 Cell viability assay

The Cell Counting Kit 8 (CCK‐8) assay (DOJINDO) was used to detect cell viability. The cells were seeded in 96‐well plates (2000 cells per well), supplemented with 10 μL CCK‐8 reagent and incubated for 2 hours. Absorbance was measured at 450 nm on a microplate reader.

### 2.8 Transwell assay

The transwell assay was conducted in 24‐well plates with 8‐µm chamber filters (Corning) to detect cell migration and invasion. A 200 μL aliquot of serum‐free cell suspension (5 × 10^5^ cells) was seeded in the upper chamber (for the invasion assay, the Matrigel was coated in advance). Then, 600 μL of culture medium (containing 10% FBS) was placed in the lower chamber. After 24 hours of incubation, the cells that penetrated the lower side of the membrane were stained with Giemsa. The migrated and invaded cells were counted by optical microscopy.

### 2.9 Tumour growth assay in nude mice

BALB/c nude mice were fed according to a protocol approved by the Ethics Committee of Shengjing Hospital. The mice were randomly divided into three groups (Vector, shRNA1 and shRNA2). Glioma cells transfected with sh‐linc00475 or shRNA were injected with 0.1 mL into the anterior right flank for the subcutaneous model. We measured tumour nodules every 5 days and executed the mice 45 days after injection. Then, the tumours were dissected and weighted. We used the formula *V* (cm^3^) = 0.5 × (length × width^2^) to calculate tumour volume. The same transfected glioma cells were injected into the right striatum of the mice for orthotopic transplantation, and the number of surviving mice was recorded every day.

### 2.10 Statistical analysis

Data are presented as mean ± SD and analysed using GraphPad Prism 7. One‐way analysis of variance, the Kaplan‐Meier method, or Student's *t* test was carried out to detect significant differences between groups. A *P*‐value < .05 was considered significant.

## 3 RESULTS

### 3.1 Linc00475 is over‐expressed in glioma tissues and cells

**Figure 1:**
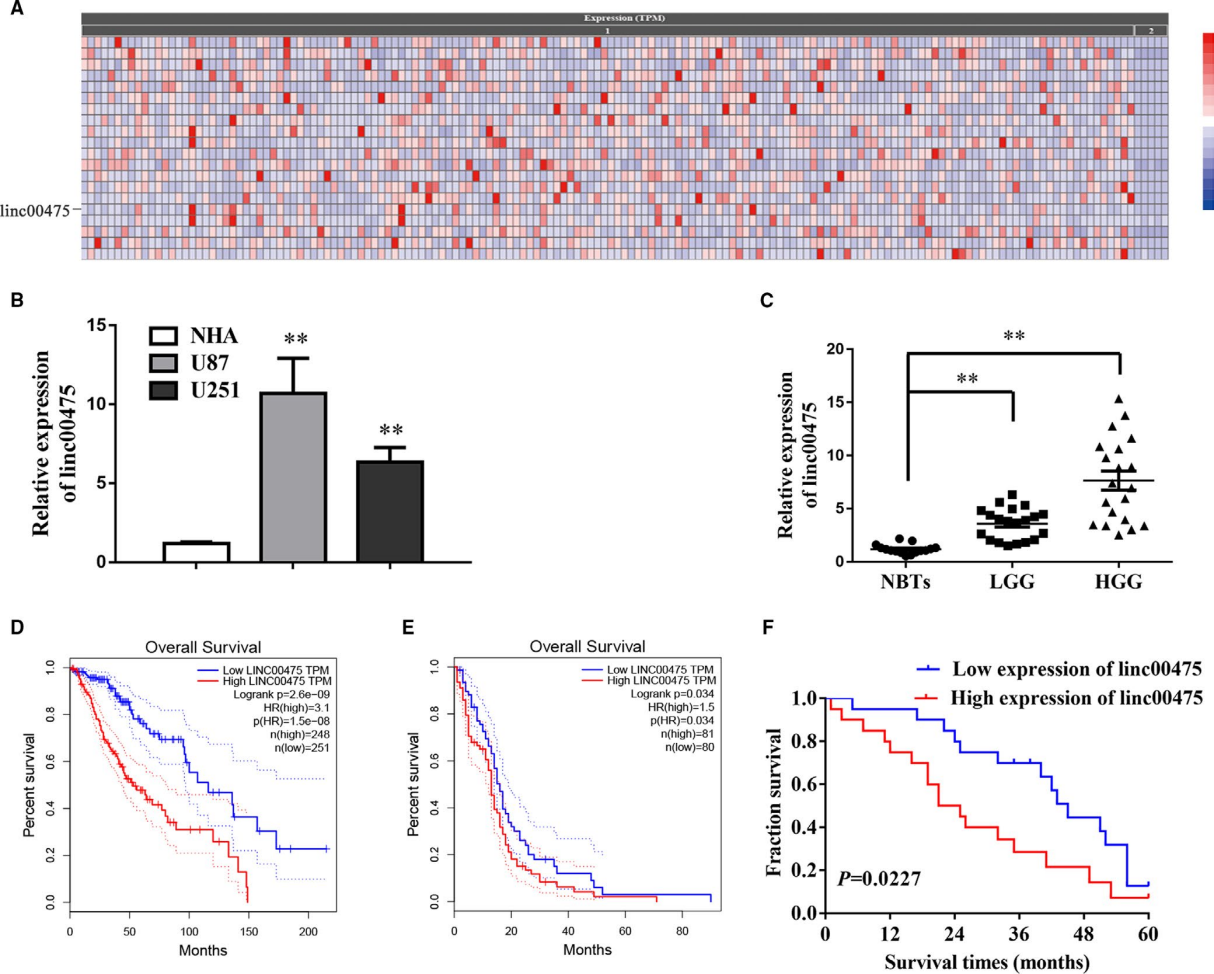
Linc00475 is over‐expressed in glioma tissues and cells. A, Linc00475 is one of the most up‐regulated lncRNAs in glioma according to the TCGA datasets (Cancer RNA‐Seq Nexus). Columns refer to the samples and rows refer to up‐regulated lncRNAs. The datasets includes 156 glioma samples (1) and 5 normal tissues (2), *P* < .01. B, qRT‐PCR analysis of linc00475 expression in normal human astrocytes (NHA) and glioma cells (U87 and U251). **P < .01 vs NHA. C, Expression of linc00475 in 15 cases of normal brain tissues (NBTs), 20 cases of low‐grade glioma (LGG) and 20 cases of high grade glioma (HGG). **P < .01 vs NBTs. D and E, Over‐expressed linc00475 was associated with shorter OS in LGG (D) and Glioblastoma (E) (GEPIA). F, Overall Kaplan–Meier survival curve for a cohort of 40 glioma patients, according to the linc00475 expression levels. The linc00475 RNA levels were normalized to those of GAPDH

LncRNAs have been confirmed to play significant roles in the regulation of many cancers, including glioma. To explore the changes of lncRNAs in glioma, we analysed The Cancer Genome Atlas (TCGA) datasets and linc00475 emerged as one of the most up‐regulated lncRNAs in glioma (Figure 1A).^22^ Then, we detected the expression of linc00475 in a cohort of 40 glioma tissues and glioma cells by qRT‐PCR. As predicted, linc00475 in glioma tissues was observed to be triple the levels of normal brain tissues (NBTs) (Figure 1B,C). Furthermore, the analysis of overall survival (OS) from Gene Expression Profiling Interactive Analysis (GEPIA) indicated that glioma patients with amplified linc00475 had shorter OS for both low‐grade glioma (LGG) and glioblastoma (GBM) (Figure 1D,E).^23^ Consistently, our survival analysis of 40 glioma patients showed a similar trend (Figure 1F).

### 3.2 Linc00475 promotes the progression of glioma cells in vitro

**Figure 2:**
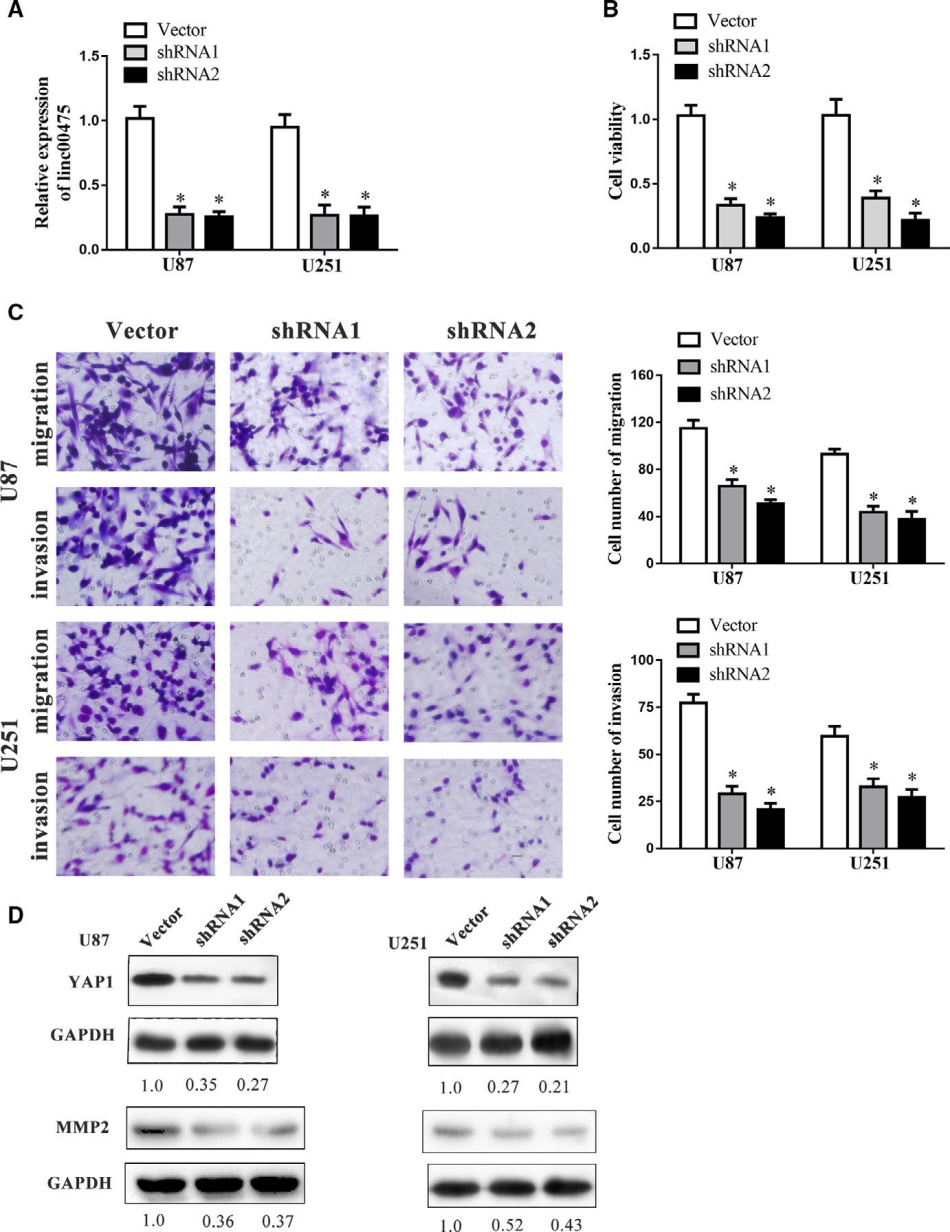
Linc00475 promotes the progression of glioma cells in vitro. A, Expression of linc00475 in glioma cells after transfection with sh‐linc00475 or the vectors. B, Cell viability of glioma cells was assessed by the CCK‐8 assay after transfection with sh‐linc00475 or the vectors. C, Effect of linc00475 on migration and invasion of glioma cells. D, Western blot analysis of YAP1 and MMP2 expression in the glioma cell lines after transfection with sh‐linc00475. For A‐C, variables are shown as mean ± SD. (n = 3, each group), *P < .05 vs Vector group. Scale bars represent 20 μm

Given the elevated expression of linc00475 in glioma and its association with shorter OS, we further explored the effect of linc00475 in glioma cells. We synthesized two target siRNAs to establish the glioma cells with suppressed linc00475. After validating transfection efficiency, we performed the CCK‐8 and transwell assays to determine cell viability, migration and invasion (Figure 2A). As predicted, knock‐down of linc00475 repressed the proliferation of glioma cells (Figure 2B). Similarly, sh‐linc00475 significantly limited the migration and invasion ability of glioma cells (Figure 2C). Matrix Metalloproteinase 2 (MMP2), which is involved in regulating the extracellular matrix, plays significant roles in glioma migration and invasion.^24^ We detected the expression of MMP2 and found that it decreased in sh‐linc00475 glioma cells (Figure 2D).

### 3.3 Restrained linc00475 inhibits glioma growth in vivo

**Figure 3:**
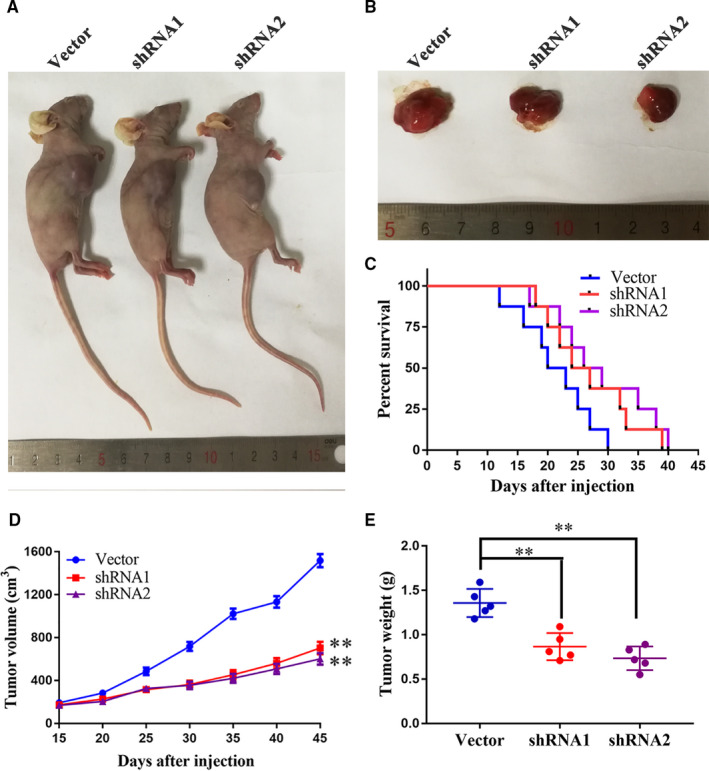
Knock‐down of linc00475 inhibits glioma growth in vivo. A and B, representative images of nude mice bearing transplant tumors (A) and sample tumors (B) from different treatment groups are shown. C, Survival curves of orthotopic xenograft from the different treatment groups (*P* > .05 vs Vector, n = 8, each group). D and E, The volume (D) and weight (E) of the harvested tumors; variables are shown as mean ± SD. (n = 5, each group), **P < .01 vs Vector group

To further confirm the oncogenesis of linc00475 in glioma, we prepared subcutaneous tumour models and orthotopic xenografts. The tumours in the shRNA1 or shRNA2 groups grew smaller compared with those in the Vector group (Figure 3A,B,D,E). Additionally, inhibiting linc00475 prolonged the survival time of the orthotopic xenografts (Figure 3C). These findings illuminate that knock‐down of linc00475 inhibited glioma in vivo.

### 3.4 Linc00475 binds directly to miR‐141‐3p

**Figure 4:**
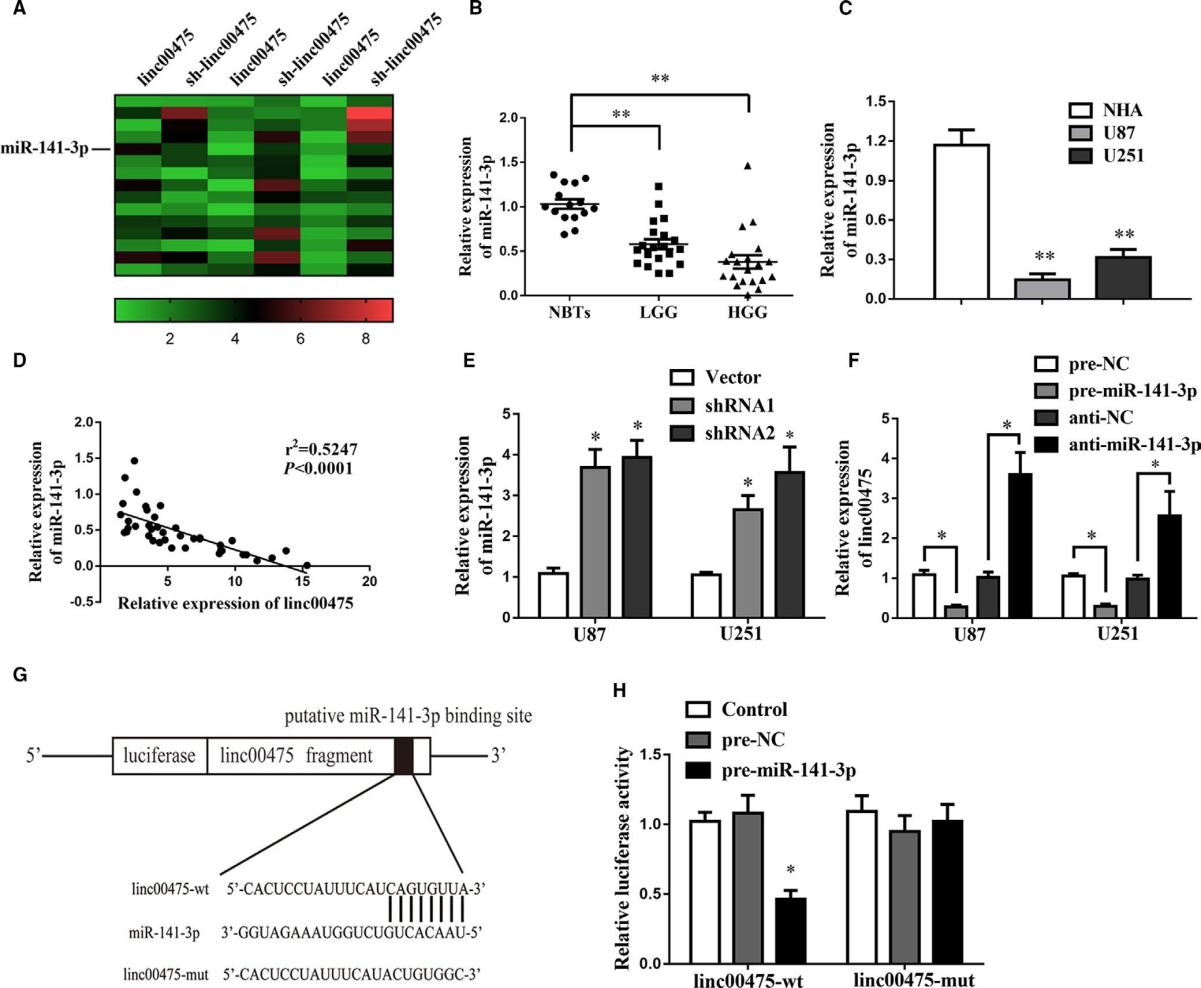
Linc00475 binds with miR‐141‐3p directly. A, Heatmap of up‐regulated miRNAs after inhibiting linc00475 in U87 glioma cells. B, Expression of miR‐141‐3p in 15 cases of NBTs, 20 cases of LGG and 20 cases of HGG. **P < .01. C, qRT‐PCR analysis of miR‐141‐3p expression in NHA and glioma cells. **P < .01 vs NHA group. D, Linear regression analysis between linc00475 and miR‐141‐3p expression levels in a cohort of 40 glioma tissues. E, Expression of miR‐141‐3p in glioma cells after transfection with sh‐linc00475. *P < .05 vs Vector. F, Expression of linc00475 in glioma cells after changing the expression of miR‐141‐3p. *P < .05. G, The luciferase reporter vectors and the predicted binding sites between linc00475 and miR‐141‐3p. H, Relative luciferase activity in HEK293T cells for the indicated treatments. *P < .05 vs pre‐NC. Variables are shown as mean ± SD (n = 3, each group)

In recent decades, competing endogenous RNA (ceRNA) has been considered as an important mechanism for lncRNA to regulate cancer progression.^25^ The oncogenic role of linc00475 prompted us to further delineate the underlying molecular mechanism. We globally analysed the change in miRNAs after knock‐down of linc00475 in glioma cells and discovered that miR‐141‐3p increased in sh‐linc00475 glioma cells (Figure 4A). A previous research reported that miR‐141‐3p was dysregulated and restrains the proliferation of glioma cells.^12^ Similarly, we confirmed that miR‐141‐3p was minimally expressed in glioma tissues and cells (Figure 4B,C). Additionally, we investigated the correlation between linc00475 and miR‐141‐3p in a cohort of 40 glioma tissues. As expected, the expression of linc00475 was negatively correlated with that of miR‐141‐3p according to Spearman's correlation analysis (Figure 4D). Furthermore, we confirmed a negative correlation between miR‐141‐3p and linc00475 in both sh‐linc00475 and anti‐miR‐141‐3p glioma cells. (Figure 4E,F). Then, we searched the Lncbase and found predictive binding sites between miR‐141‐3p and linc00475 (Figure 4G). A dual‐luciferase reporter gene assay further validated the responsible binding sites of miR‐141‐3p and linc00475 (Figure 4H).

### 3.5 MiR‐141‐3p directly targets the YAP1‐3′UTR and reduces its expression

**Figure 5:**
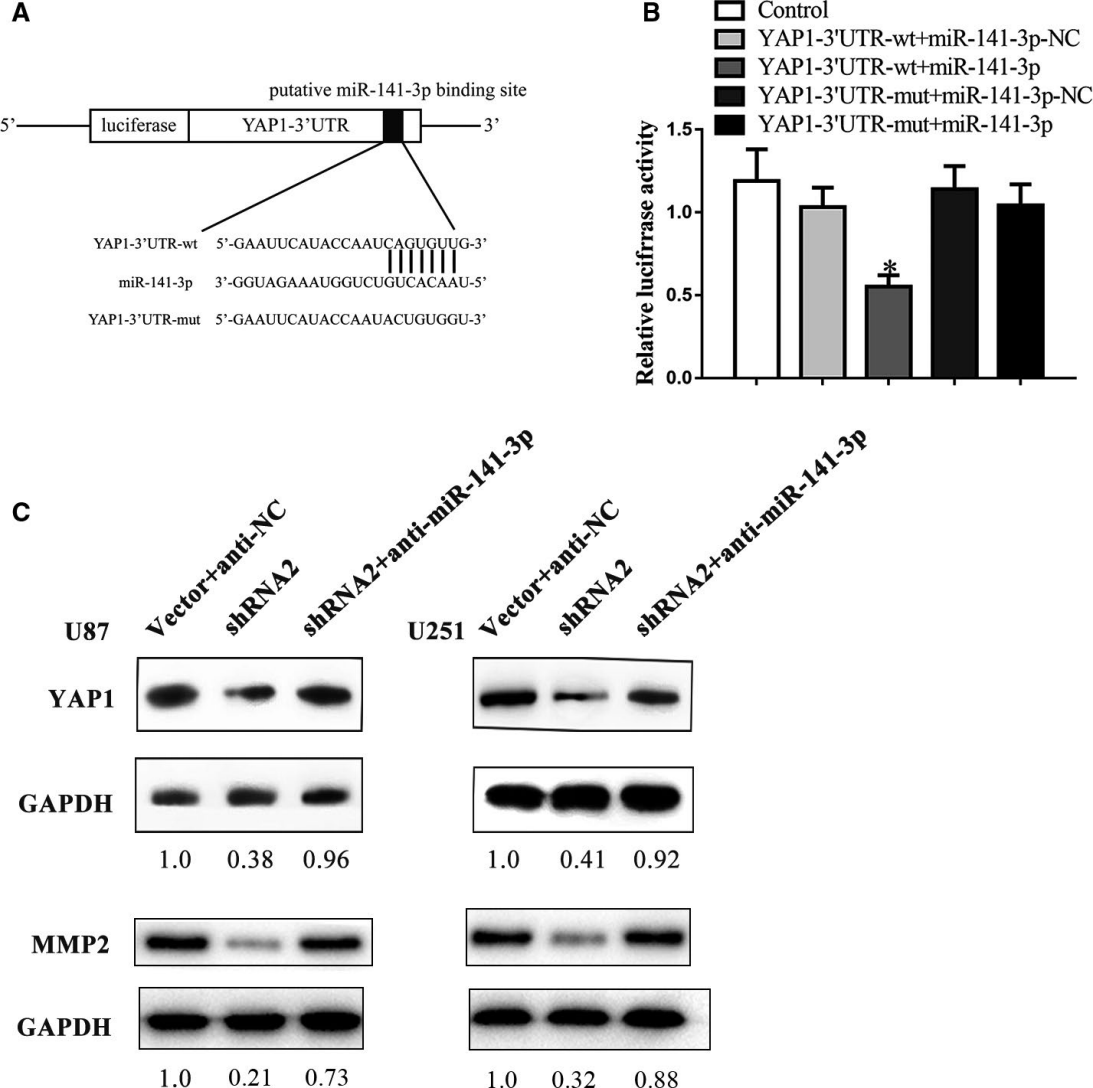
MiR‐141‐3p binds with the YAP1‐3′UTR directly. A, The luciferase reporter vectors and the predicted binding sites between YAP1‐3′UTR and miR‐141‐3p. B, Relative luciferase activity for the indicated treatments. Variables are shown as mean ± SD. (n = 3, each group) *P < .05 vs YAP‐1‐3′UTR‐mut + miR‐141‐3p. C, The Western blot analysis uncovers that inhibiting miR‐141‐3p restores the shRNA2‐induced restrain of YAP1 and MMP2

Numerous recent studies have shown that miRNAs regulate cancer progression by interacting with untranslated regions (UTRs) of mRNA in molecular pathways.^26^ The binding sites between the YAP1 3'‐UTR and miR‐141‐3p were predicted based on the ENCORI database. We conducted the dual‐luciferase assay to ascertain the interaction between miR‐141‐3p and YAP1. The results revealed that the YAP1‐3’UTR‐wt + miR‐141‐3p group presented minimum relative luciferase activity (Figure 5A,B).

YAP1 is a key effector of the Hippo pathway and has emerged as a significant determinant of malignancy in glioma.^27^ The Western blot analysis was carried out to identify the roles of YAP1 in the regulation of the linc00475/miR‐141‐3p axis. Expectedly, the expression of YAP1 was down‐regulated after knock‐down of linc00475 in glioma cells (Figure 2D). YAP1 and MMP2 expression levels restored in cells co‐transfected with sh‐linc00475 and anti‐miR‐141‐3p. (Figure 5C).

### 3.6 Linc00475 facilitates glioma progression by regulating miR‐141‐3p/YAP1

**Figure 6:**
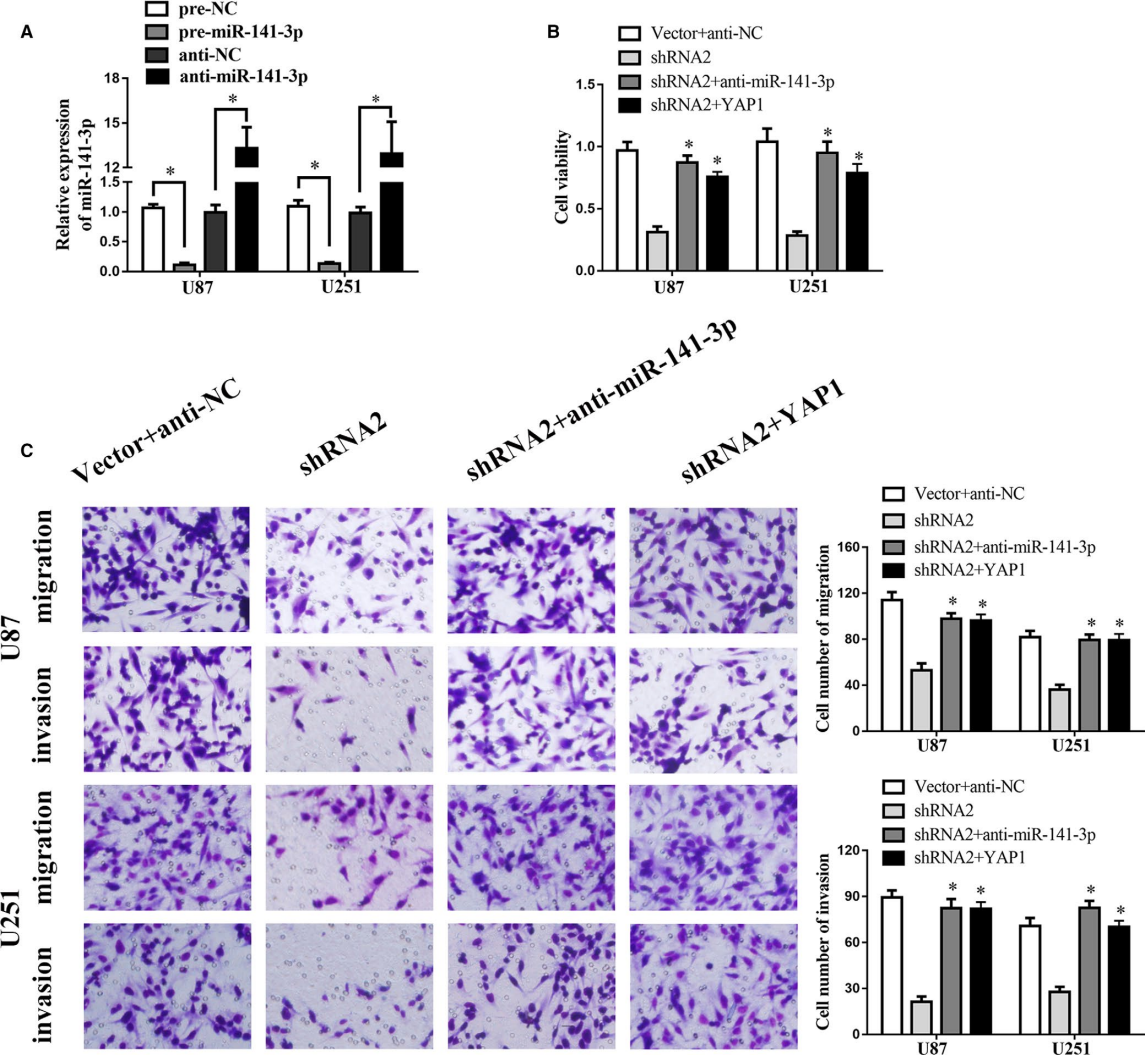
Linc00475 facilitates the progression of glioma by regulating miR‐141‐3p/YAP1. A, The expression of miR‐141‐3p measured by qPCR in over‐expressed or silenced miR‐141‐3p glioma cells.*P < .05. B, Viability of glioma cells via CCK‐8 assays with sh‐linc00475 was rescued by co‐transfection with shRNA2 and anti‐miR‐141‐3p or co‐transfection with shRNA2 and YAP1. C, Migration and invasion of glioma cells via transwell assays with sh‐linc00475 was rescued by co‐transfection with shRNA2 and anti‐miR‐141‐3p or co‐transfection with shRNA2 and YAP1. Variables are shown as mean ± SD. (n = 3, each group), *P < .05 vs shRNA2. Scale bars represented 20 μm

Results above indicate that linc00475 binds with miR‐141‐3p, and miR‐141‐3p binds directly to the YAP1‐3′UTR. We performed rescue assays to confirm whether miR‐141‐3p and YAP1 are involved in linc00475‐mediated glioma development. After determining transfection efficiency (Figure 6A and Figure S1), we performed the CCK‐8 and transwell assays. As seen in Figure 6B, the cell viability limited by sh‐linc00475 was rescued by sh‐linc00475 + anti‐miR‐141‐3p and sh‐linc00475 + YAP1. Furthermore, the transwell assays indicated the same trend (Figure 6C).

## 4 DISCUSSION

This current study revealed that the expression of linc00475 elevates in glioma. In addition, we demonstrated that high linc00475 expression predicted shorter survival in patients suffering from glioma. Similarly, in vivo experiments indicated that inhibiting linc00475 restrained the proliferation and prolonged the survival time of xenografts. Furthermore, we identified that the tumorigenesis of linc00475 occurred via the miR‐141‐3p/YAP1 pathway. These findings reveal that the expression of linc00475 may be a key biomarker for evaluating the prognosis of patients with glioma, highlighting the potential role of linc00475 to serve as a therapeutic target. Accumulating evidence during the last decade has suggested that lncRNAs play functional roles in cancers.^28^, ^29^ Furthermore, many lncRNAs play complex and extensive roles in the malignant progression of glioma.^30^, ^31^, ^32^ In this research, we retrieved TCGA datasets and recognized linc00475 as one of the most up‐regulated lncRNAs in glioma. Then, we confirmed that high linc00475 expression predicted a poor OS in patients with glioma. Moreover, we discovered that inhibiting linc00475 attenuated tumorigenesis of glioma in vitro and in vivo. However, we used an empty vector as the negative control for the shRNAs, which was an oversight that needs to be corrected in a future study.

Many studies have described the role of miR‐141‐3p in cancers. MiR‐141‐3p is reportedly dysregulated and indicates a poor outcome in patients with hepatocellular carcinoma.^33^ Over‐expressing miR‐141‐3p restrains the tumorigenesis of colorectal cancer cells by regulating TRAF5.^34^ Furthermore, miR‐141‐3p is determined to be highly expressed in exosomes of plasma from patients’ with rectal cancer accompanied by liver metastasis, showing the value of metastatic prediction.^35^ MiR‐141‐3p is report to express at low levels in glioma and hinders the malignant growth of glioma cells in vitro.^36^ Our study proved that miR‐141‐3p was expressed at low levels in glioma and was involved in the regulation of linc00475 by acting as a sponge for YAP1.

Recently, the roles of the Hippo/YAP1 pathway in oncogenic transformation have aroused researchers’ attention. Extensive studies have discovered that YAP1 promotes the transcriptional activity of p53.^37^ Amplifying YAP1 leads to the acquisition of oncogenic cell features, including rapid proliferation, migration and invasion in breast cancer.^38^ YAP1 has been confirmed to be account for therapeutic resistance in lung cancer.^39^ YAP1 promotes cell competition in glioma by decreasing apoptosis, and knock‐down of YAP1 attenuates self‐renewal capability.^15^, ^40^ This study illustrates that miR‐141‐3p inhibits tumorigenesis of glioma by targeting YAP1.

Overall, linc00475 was significantly up‐regulated in glioma, and patients with amplified linc00475 had a shorter OS. Knock‐down of linc00475 inhibited tumorigenesis of glioma through the miR‐141‐3p/YAP1 pathway. Our study provides new avenues for therapeutic targets of glioma.

## CONFLICTS OF INTEREST

The authors declare that they have no competing interests.

## AUTHOR CONTRIBUTION

**Mingjun Yu:** Data curation (equal); Funding acquisition (equal); Investigation (lead); Writing‐original draft (lead); Writing‐review & editing (equal). **Bolong Yi:** Formal analysis (equal); Methodology (lead); Writing‐review & editing (equal). **Wen Zhou:** Formal analysis (equal); Software (equal); Writing‐review & editing (equal). **Wei Gong:** Data curation (supporting); Investigation (equal); Methodology (equal). **Gang Li:** Formal analysis (equal); Methodology (equal); Software (equal). **Shijia Yu:** Data curation (equal); Funding acquisition (equal); Supervision (lead); Writing‐review & editing (equal).

## ETHICAL APPROVAL AND INFORM CONSENT

The study not only received informed consent from all patients, but also received the support of the ethics committee of Shengjing Hospital of China Medical University.

## Supporting information

Fig S1

Table S1

Table S2

## Data Availability

All data generated or analysed during this study are included in this article.

## References

[1] Zhang LE , Liu Y , Wang M , et al. EZH2‐, CHD4‐, and IDH‐linked epigenetic perturbation and its association with survival in glioma patients. J Mol Cell Biol. 2017;9:477‐488. 29272522 10.1093/jmcb/mjx056 PMC5907834

[2] Li Y , Xu J , Zhang J , et al. MicroRNA‐346 inhibits the growth of glioma by directly targeting NFIB. Cancer Cell Int. 2019;19:294. 31807116 10.1186/s12935-019-1017-5 PMC6857291

[3] Wang Y , Sun L , Wang L , et al. Long non‐coding RNA DSCR8 acts as a molecular sponge for miR‐485‐5p to activate Wnt/beta‐catenin signal pathway in hepatocellular carcinoma. Cell Death Dis. 2018;9:851. 30154476 10.1038/s41419-018-0937-7 PMC6113322

[4] Yu H , Zheng J , Liu X , et al. Transcription factor NFAT5 promotes glioblastoma cell‐driven angiogenesis via SBF2‐AS1/miR‐338‐3p‐mediated EGFL7 expression change. Front Mol Neurosci. 2017;10:301. 28983240 10.3389/fnmol.2017.00301 PMC5613209

[5] Liu B , Cao W , Ma H . Knockdown of lncRNA LSINCT5 suppresses growth and metastasis of human glioma cells via up‐regulating miR‐451. Artif Cells Nanomed Biotechnol. 2019;47:2507‐2515. 31213092 10.1080/21691401.2019.1626404

[6] Long S , Li G . Comprehensive analysis of a long non‐coding RNA‐mediated competitive endogenous RNA network in glioblastoma multiforme. Exp Ther Med. 2019;18:1081‐1090. 31316603 10.3892/etm.2019.7647 PMC6601370

[7] Ye J , Tan L , Fu YU , et al. LncRNA SNHG15 promotes hepatocellular carcinoma progression by sponging miR‐141‐3p. J Cell Biochem. 2019;120:19775‐19783. 31310393 10.1002/jcb.29283

[8] Liu Y , Zhao R , Wang H , et al. miR‐141 is involved in BRD7‐mediated cell proliferation and tumor formation through suppression of the PTEN/AKT pathway in nasopharyngeal carcinoma. Cell Death Dis. 2016;7:e2156. 27010857 10.1038/cddis.2016.64 PMC4823963

[9] Yao Y‐S , Qiu W‐S , Yao R‐Y , et al. miR‐141 confers docetaxel chemoresistance of breast cancer cells via regulation of EIF4E expression. Oncol Rep. 2015;33:2504‐2512. 25813250 10.3892/or.2015.3866

[10] Wang J , Wang G , Li B , et al. miR‐141‐3p is a key negative regulator of the EGFR pathway in osteosarcoma. OncoTargets Ther. 2018;11:4461‐4478. 10.2147/OTT.S171304 PMC6074763 30104888

[11] Guo E , Wang Z , Wang S . MiR‐200c and miR‐141 inhibit ZEB1 synergistically and suppress glioma cell growth and migration. Eur Rev Med Pharmacol Sci. 2016;20:3385‐3391. 27608897

[12] Wang M , Hu M , Li Z , et al. miR‐141‐3p functions as a tumor suppressor modulating activating transcription factor 5 in glioma. Biochem Biophys Res Commun. 2017;490:1260‐1267. 28595907 10.1016/j.bbrc.2017.05.179 PMC5759330

[13] Kandasamy S , Adhikary G , Rorke EA , et al. The YAP1 signaling inhibitors, verteporfin and CA3, suppress the mesothelioma cancer stem cell phenotype. Mol Cancer Res. 2019;18(3):343–351. 31732616 10.1158/1541-7786.MCR-19-0914 PMC7064165

[14] Guichet PO , Masliantsev K , Tachon G , et al. Fatal correlation between YAP1 expression and glioma aggressiveness: clinical and molecular evidence. J Pathol. 2018;246:205‐216. 30009411 10.1002/path.5133

[15] Liu Z , Yee PP , Wei Y , et al. Differential YAP expression in glioma cells induces cell competition and promotes tumorigenesis. J Cell Sci. 2019;132:jcs225714. 30665893 10.1242/jcs.225714 PMC6432718

[16] Li Z , Ge X , Lu J , et al. MiR‐141‐3p regulates proliferation and senescence of stem cells from apical papilla by targeting YAP. Exp Cell Res. 2019;383:111562. 31437458 10.1016/j.yexcr.2019.111562

[17] Yu M , Xue Y , Zheng J , et al. Linc00152 promotes malignant progression of glioma stem cells by regulating miR‐103a‐3p/FEZF1/CDC25A pathway. Mol Cancer. 2017;16:110. 28651608 10.1186/s12943-017-0677-9 PMC5485714

[18] Yu S , Yu M , He X , et al. KCNQ1OT1 promotes autophagy by regulating miR‐200a/FOXO3/ATG7 pathway in cerebral ischemic stroke. Aging Cell. 2019;18:e12940. 30945454 10.1111/acel.12940 PMC6516167

[19] Yu M , Yu S , Xue Y , et al. Over‐expressed FEZF1 predicts a poor prognosis in glioma and promotes glioma cell malignant biological properties by regulating Akt‐ERK pathway. J Mol Neurosci. 2018;65:411‐419. 30030762 10.1007/s12031-018-1108-0

[20] Paraskevopoulou MD , Vlachos IS , Karagkouni D , et al. DIANA‐LncBase v2: indexing microRNA targets on non‐coding transcripts. Nucleic Acids Res. 2016;44:D231‐D238. 26612864 10.1093/nar/gkv1270 PMC4702897

[21] Li J‐H , Liu S , Zhou H , et al. starBase v2.0: decoding miRNA‐ceRNA, miRNA‐ncRNA and protein‐RNA interaction networks from large‐scale CLIP‐Seq data. Nucleic Acids Res. 2014;42:D92‐D97. 24297251 10.1093/nar/gkt1248 PMC3964941

[22] Li J‐R , Sun C‐H , Li W , et al. Cancer RNA‐Seq Nexus: a database of phenotype‐specific transcriptome profiling in cancer cells. Nucleic Acids Res. 2016;44:D944‐D951. 26602695 10.1093/nar/gkv1282 PMC4702907

[23] Tang Z , Li C , Kang B , et al. GEPIA: a web server for cancer and normal gene expression profiling and interactive analyses. Nucleic Acids Res. 2017;45:W98‐W102. 28407145 10.1093/nar/gkx247 PMC5570223

[24] Zhou W , Yu X , Sun S , et al. Increased expression of MMP‐2 and MMP‐9 indicates poor prognosis in glioma recurrence. Biomed Pharmacother. 2019;118:109369. 31545229 10.1016/j.biopha.2019.109369

[25] Zong Z , Song Y , Xue Y , et al. Knockdown of LncRNA SCAMP1 suppressed malignant biological behaviours of glioma cells via modulating miR‐499a‐5p/LMX1A/NLRC5 pathway. J Cell Mol Med. 2019;23:5048‐5062. 31207033 10.1111/jcmm.14362 PMC6653555

[26] Li W , Wang L , Ji X‐B , et al. MiR‐199a inhibits tumor growth and attenuates chemoresistance by targeting K‐RAS via AKT and ERK signalings. Front Oncol. 2019;9:1071. 31681604 10.3389/fonc.2019.01071 PMC6803549

[27] Melhuish TA , Kowalczyk I , Manukyan A , et al. Myt1 and Myt1l transcription factors limit proliferation in GBM cells by repressing YAP1 expression. Biochim Biophys Acta Gene Regul Mech. 2018;1861:983‐995. 30312684 10.1016/j.bbagrm.2018.10.005 PMC6203443

[28] Ma Y , Yang Y , Wang F , et al. Long non‐coding RNA CCAL regulates colorectal cancer progression by activating Wnt/beta‐catenin signalling pathway via suppression of activator protein 2alpha. Gut. 2016;65:1494‐1504. 25994219 10.1136/gutjnl-2014-308392

[29] Lingadahalli S , Jadhao S , Sung YY , et al. Novel lncRNA LINC00844 regulates prostate cancer cell migration and invasion through AR signaling. Mol Cancer Res. 2018;16:1865‐1878. 30115758 10.1158/1541-7786.MCR-18-0087

[30] Zheng Y‐J , Liang T‐S , Wang J , et al. Silencing lncRNA LOC101928963 inhibits proliferation and promotes apoptosis in spinal cord glioma cells by binding to PMAIP1. Mol Ther Nucl Acids. 2019;18:485‐495. 10.1016/j.omtn.2019.07.026 PMC6838552 31670198

[31] Zhu J , Gu W , Yu C . MATN1‐AS1 promotes glioma progression by functioning as ceRNA of miR‐200b/c/429 to regulate CHD1 expression. Cell Prolif. 2020;53:e12700. 31667976 10.1111/cpr.12700 PMC6985690

[32] Liu X , Zheng J , Xue Y , et al. PIWIL3/OIP5‐AS1/miR‐367‐3p/CEBPA feedback loop regulates the biological behavior of glioma cells. Theranostics. 2018;8:1084‐1105. 29464001 10.7150/thno.21740 PMC5817112

[33] Hou XU , Yang LE , Jiang X , et al. Role of microRNA‐141‐3p in the progression and metastasis of hepatocellular carcinoma cell. Int J Biol Macromol. 2019;128:331‐339. 30695725 10.1016/j.ijbiomac.2019.01.144

[34] Liang Z , Li X , Liu S , et al. MiR‐141‐3p inhibits cell proliferation, migration and invasion by targeting TRAF5 in colorectal cancer. Biochem Biophys Res Commun. 2019;514:699‐705. 31078266 10.1016/j.bbrc.2019.05.002

[35] Meltzer S , Bjørnetrø T , Lyckander LG , et al. Circulating exosomal miR‐141‐3p and miR‐375 in metastatic progression of rectal cancer. Transl Oncol. 2019;12:1038‐1044. 31146167 10.1016/j.tranon.2019.04.014 PMC6542769

[36] Peng T , Zhang S , Li W , et al. MicroRNA‐141 inhibits glioma cells growth and metastasis by targeting TGF‐beta2. Am J Transl Res. 2016;8:3513‐3521. 27648141 PMC5009403

[37] Rivas S , Anton IM , Wandosell F . WIP‐YAP/TAZ as a new pro‐oncogenic pathway in glioma. Cancers. 2018;10(6):191. 10.3390/cancers10060191 PMC6024887 29890731

[38] Chan SW , Lim CJ , Guo K , et al. A role for TAZ in migration, invasion, and tumorigenesis of breast cancer cells. Cancer Res. 2008;68:2592‐2598. 18413727 10.1158/0008-5472.CAN-07-2696

[39] Gobbi G , Donati B , Do Valle IF , et al. The Hippo pathway modulates resistance to BET proteins inhibitors in lung cancer cells. Oncogene. 2019;38:6801‐6817. 31406246 10.1038/s41388-019-0924-1

[40] Yu OM , Benitez JA , Plouffe SW , et al. YAP and MRTF‐A, transcriptional co‐activators of RhoA‐mediated gene expression, are critical for glioblastoma tumorigenicity. Oncogene. 2018;37:5492‐5507. 29887596 10.1038/s41388-018-0301-5 PMC6195840

