# Supplementary figures and images for: Linc00475 promotes the progression of glioma by regulating the miR‐141‐3p/YAP1 axis
Source: J Cell Mol Med. 2020 Dec 18;25(1):463–72. doi: 10.1111/jcmm.16100 (PMC7810941; doi:10.1111/jcmm.16100)

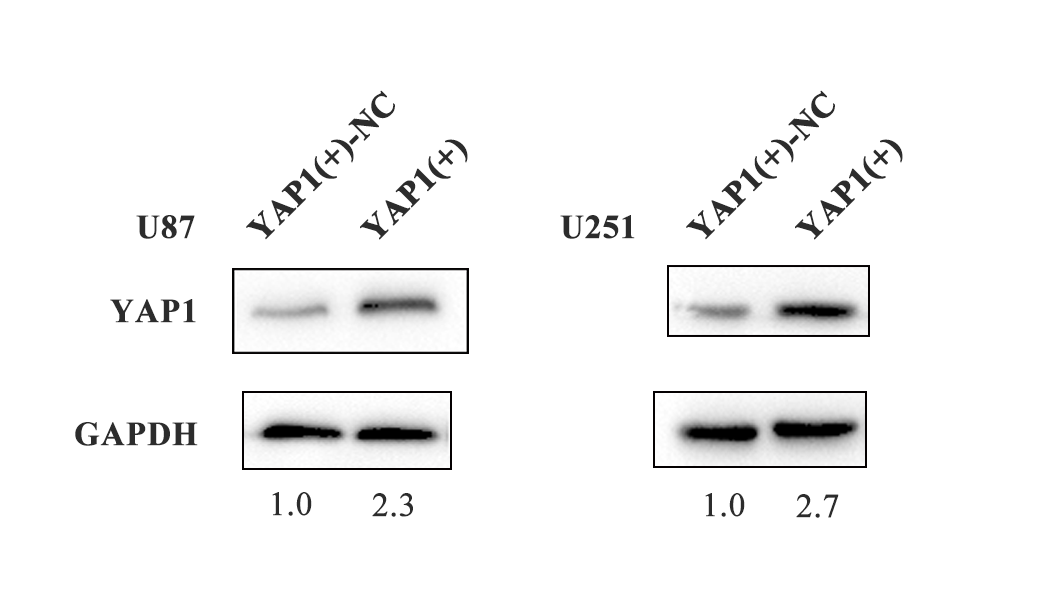

Supplement: Supplementary file 1 — Fig S1 [file JCMM-25-463-s001.tif]
