# Supplementary material for: Linc00475 promotes the progression of glioma by regulating the miR‐141‐3p/YAP1 axis
Source: J Cell Mol Med. 2020 Dec 18;25(1):463–72. doi: 10.1111/jcmm.16100 (PMC7810941; doi:10.1111/jcmm.16100)
Supplement: Supplementary file 2 — Table S1 [file JCMM-25-463-s002.docx]

Table S1: clinical characteristics of the patients:

| Patient ID | Age | Pathological grade | Death  (1=yes, 0=no) | OS.m |
| --- | --- | --- | --- | --- |
| Case1  Case2  Case3  Case4  Case5  Case6  Case7  Case8  Case9  Case10  Case11  Case12  Case13  Case14  Case15  Case16  Case17  Case18  Case19  Case20  Case21  Case22  Case23  Case24  Case25  Case26  Case27  Case28  Case29  Case30  Case31  Case32  Case33  Case34  Case35  Case36  Case37  Case38  Case39  Case40 | 52  34  45  37  41  48  39  27  71  6  65  53  49  59  73  51  66  68  42  38  62  39  3  72  6  71  75  2  55  2  56  72  63  42  39  3  49  48  57  51 | Low  Low  Low  High  Low  High  High  Low  Low  Low  High  Low  High  Low  Low  Low  High  Low  Low  Low  Low  High  High  High  High  High  Low  High  High  High  Low  Low  High  High  Low  High  High  High  Low  High | 1  1  1  1  1  0  0  1  1  0  1  1  1  1  1  0  1  1  1  1  0  1  1  1  1  1  1  1  1  1  1  1  1  1  1  1  1  0  1  1 | 51  17  40  56  52  38  35  56  56  80  5  45  43  24  25  32  22  68  32  42  35  16  41  1  25  26  3  7  35  12  49  21  11  69  53  19  19  26  32  21 |
