# Supplementary material for: Linc00475 promotes the progression of glioma by regulating the miR‐141‐3p/YAP1 axis
Source: J Cell Mol Med. 2020 Dec 18;25(1):463–72. doi: 10.1111/jcmm.16100 (PMC7810941; doi:10.1111/jcmm.16100)
Supplement: Supplementary file 3 — Table S2 [file JCMM-25-463-s003.docx]

Table S2: sequences of siRNAs:

| Gene | Sequences |
| --- | --- |
| linc00475 si1 | 5’-GCAGGCCTATTCTATGAAAGG-3’ |
| linc00475 si2 | 5’- GCATTCTCATGACTGCTTTAA-3’ |
| Negative Control siRNA | 5’-AGUACAGCAAACGAUACGGTT-3’ |
| miR-141-3p mimics | sense: 5’-UAACACUGUCUGGUAAAGAUGG-3’ |
|  | antisense: 5’- AUCUUUACCAGACAGUGUUAUU-3’ |
| miR-141-3p mimics NC | sense: 5’-UUCUCCGAACGUGUCACGUTT-3’ |
|  | antisense: 5’-ACGUGACACGUUCGGAGAATT-3’ |
| miR-141-3p inhibitor | 5’- CCAUCUUUACCAGACAGUGUUA-3’ |
| miR-141-3p inhibitor NC | 5’-UUGUACUACACAAAAGUACUG-3’ |
